# Spatial distribution of immune checkpoint proteins in histological subtypes of lung adenocarcinoma

**DOI:** 10.1016/j.neo.2021.05.005

**Published:** 2021-06-05

**Authors:** Sarah Müller, Stefanie Mayer, Peter Möller, Thomas F.E. Barth, Ralf Marienfeld

**Affiliations:** Institute of Pathology, Ulm University, Ulm, Germany

**Keywords:** NSCLC, Immune checkpoint proteins, Heterogeneity

## Abstract

The most prevalent histological type of non-small cell lung cancer (NSCLC) is adenocarcinoma. The WHO classifies this tumor into subtypes according to the predominant growth pattern such as lepidic, acinar, papillary, solid or micropapillary, each harboring specific molecular features. NSCLC adenocarcinoma heterogeneity is discussed to be a reason for therapy failure using targeted therapy or immune checkpoint inhibitors. For successful therapy of immune checkpoint inhibitors the expression and distribution of the involved immune checkpoint proteins is essential. Therefore, we aimed to investigate the distribution of five prominent immune checkpoint proteins in regard of the histological growth patterns of lung adenocarcinoma. We performed immunohistochemical staining of 84 tumor segments from 22 resected tumor samples to evaluate the expression of PD-L1, PD-1, Nectin-2, PVR, and TIGIT in distinct growth patterns of lung adenocarcinoma. We determined a distinct heterogeneity between and within different tumor segments regarding morphological growth patterns. Furthermore, expression of immune checkpoint proteins varied between different growth pattern areas as well as within one distinct growth pattern. Expression of PVR was significantly higher in solid compared to acinar growth pattern (p= 0.00736). Of note, we detected TIGIT not only on tumor infiltrating lymphocytes but also on tumor cells, whereas non-neoplastic lung tissue was consistently TIGIT-negative. The immune checkpoint protein distribution in histologic subtypes of pulmonary adenocarcinoma displays an considerable intra- and intertumoral heterogeneity implying the requirement of either a multiregion or an adjusted analysis when determining the expression status of PD-1:PD-L1 and the TIGIT:PVR/Nectin-2 checkpoint proteins as predictive markers.

## Introduction

Lung cancer is the leading cause in cancer related death worldwide [1], with non-small cell lung cancer (NSCLC) being the most frequent subtype [2], of which in turn adenocarcinoma is the most prevalent [3]. As lung adenocarcinoma is intrinsically very heterogenic, the WHO Classification of 2015 is classifying this tumor into subtypes according to the predominant growth pattern, which can be lepidic, acinar, papillary, solid or micropapillary [2]. These histological subtypes were shown to correlate with prognosis 4, 5, 6. Tumors of various entities, including NSCLC, are able to evade the immune system via upregulation of immune checkpoint molecules, representing one of multiple mechanisms circumventing immunosurveillance and promoting survival of tumor cells [7]. A prominent example of such a tumor-mediated attenuation of immune surveillance is the interaction of the inducible ligand PD-L1 (B7-H1, CD274) to its receptor PD-1, which is expressed on tumor infiltrating lymphocytes (TILs) and also tumor cells. The PD-1:PD-L1 interaction results in a bidirectional inhibitory signal which leads to an exhausted state of immune cells expressing these molecules [8]. This dysfunctional state is hallmarked by the loss of effector function, including proliferation, release of cytokines, and secretion of cytolytic factors. In return, the tumor cells themselves also show resistance towards T-cell mediated cytotoxicity [9]. To block these co-inhibitory signals and prevent inactivation of T-cells, the FDA approved the antagonistic PD-1 antibodies nivolumab (Opdivo, 2015) and pembrolizumab (Keytruda, 2015) as well as PD-L1 antibody atezolizumab (Tecentriq, 2017) as second-line therapy for advanced NSCLC with progression after platinum-based chemotherapy. Further, pembrolizumab is also approved as first-line therapy for advanced NSCLS patients with >50% PD-L1 expressing tumor cells [10]. However, response rates remain quite low, as only 20% of all patients primarily respond to therapy 11, 12, 13. Clinical trials also revealed conflicting results regarding PD-L1 status as a biomarker, which is not consistently associated with therapy response [13]. Furthermore, adaptive or acquired resistance is observed in many patients who initially exhibit effective response to PD-1/PD-L1 antibody treatment [14]. One reason for this treatment failure may be additional inhibitory immune checkpoint pathways used by the tumor as a redundancy system to control anti-tumoral immune responses [15]. These alternative pathways need to be co-targeted in order to provide a full and sustained clinical response. Recently, one of these additional immune checkpoint pathways moved into the spotlight i.e.,T cell immunoreceptor with immunoglobulin and ITIM domain (TIGIT, Vstm3, WUCAM, VSIG9) [16]. In several tumor entities, including lung adenocarcinoma, the expression of this regulatory receptor was found to be increased on TILs, giving tumor cells an additional option to escape the immune system in suppressing the functions of the TILs, when TIGIT binds one of its ligands Nectin-2 or PVR [17]. Moreover, this aberrant overexpression was correlated with poor clinical outcome [18]. So far six anti-TIGIT antibodies are being tested, either investigating the eligibility for a mono-anti-TIGIT-therapy or as part of combination therapies to synergize with either PD-1, PD-L1 or CTLA-4 blockade [16]. It was demonstrated that dual PD-1/TIGIT blockade potently increases tumor antigen-specific CD8+ T cell expansion and function in vitro and promotes tumor rejection in mouse tumor models leading to a cure rate up to 100% [16].

Given the pivotal role of the immune checkpoints PD-L1:PD-1 and TIGIT:PVR/Nectin-2 for the immune evasion of NSCLC adenocarcinoma and for the treatment success using immune checkpoint inhibitors, our aim was to dissect the heterogeneity of the expression of these immune checkpoint proteins in NSCLC adenocarcinoma. Here, we focused especially on the heterogeneity in conjunction with the different NSCLC adenocarcinoma growth patterns.

## Material & methods

### Patient and tumor characteristics

22 patients were enrolled in this study. All patients were required to meet the criteria: (A) the patients had to be diagnosed with NSCLC adenocarcinoma, (B) tumor resection was performed and (C) archived FFPE tissue of the resected tumor with sufficient tumor tissue was available. This results in 84 tumor segments derived from this cohort. Resected human tumor tissue used in the current study were collected and stored by the Institute of Pathology of the University Medical Centre Ulm. Pathologists assessed all samples before use. The study was approved by the ethics committee of the University of Ulm (ethic code 180/19) and is in line with the declaration of Helsinki.

### Immunohistochemistry

Immunohistochemistry was performed on serial 2 µm-thick tissue sections cut from formalin-fixed, paraffin-embedded tissue blocks. Staining was performed according to standardized protocols using the commercially available Dako REAL detection system (Dako, Santa Clara, USA) and the Vectastain Elite Kit (Vector Laboratories, Burlingame, USA). Briefly, all slides were deparaffinized in xylene and underwent a series of incubations in decreasing ethanol concentrations for rehydration. Antigen retrieval was performed using different treatments specific for each antibody, including steaming (PD-L1, Nectin-2) and microwaving (PVR, PD1 and TIGIT) in different buffer solutions, EDTA buffer pH 9.0 (PD-L1, Nectin-2), citrate buffer pH 6.0 (PD-1, PVR) or TRIS-based buffer pH 9.0 (TIGIT) for 20 min. Incubation with the primary antibody was carried out for 30 min at room temperature (PD-L1, Nectin-2, PVR, PD-1) or overnight for at least 16h at 4°C (TIGIT). Further, sections were counterstained with hematoxylin.

### Antibodies

The following antibodies were used: monoclonal antibody against PD-L1 (Quartett, Berlin, Germany, 1:200, QR1), PD-1 (Dianova, Hamburg, Germany, 1:50, JAD1), Nectin-2 (Cell Signaling, Danvers, USA, 1:50, D8D3F), PVR (Cell Signaling, Danvers, USA, 1:50, D8A5G), and TIGIT (Dianova, Hamburg, Germany, 1:25, TG1).

### Transfection of HEK-293 cells

To test the specificity of the anti-TIGIT antibody immunohistochemistry stainings were carried out using human embryonic kidney cells 293 (HEK-293) which ectopically express human TIGIT. For this purpose HEK-293 cells were grown in IMDM/RPMI (4:1) supplemented with 10% fetal bovine serum, glutamine, 100 U/ml penicillin, and 100 μg/ml streptomycin at 37°C in a humified atmosphere with 5% CO_2_, reagents were purchased from Lonza (Basel, Switzerland) and Biochrom (Harvard Bioscience, Hill Road Holliston, USA). HEK-293 cells were transfected via the calcium-phosphate method as described previously [19]. HEK293-TIGIT cells were generated by transfecting a TIGIT expression vector pcDNA3.1+/C-(K)DYK (concentration 0.1 μg/μL, GenEZ^TM^ ORF clone, GenScript, New Jersey, USA) and the pcDNA3.1 empty vector (eV) serving as a control. After transfection, cells were pelleted and fixed in buffered 4% formalin solution (Langenbrinck, Emmendingen, Germany) for 24 hours, dehydrated and embedded in paraffin. Subsequently, 2 μm-thick paraffin sections of the cellblocks were subjected to immunocytological staining (see “Immunohistochemistry”).

### Evaluation of immunohistochemical staining

The distinct histological growth patterns in each segment were determined by an experienced pathologist on consecutive H&E-stained sections; the pathologist himself was blinded from clinical information. In each segment the available histological growth patterns were determined as lepidic, acinar, papillary, solid, and micropapillary, with up to 4 different growth patterns in one segment. Subsequently, molecular and histopathological examinations were carried out separately for each growth pattern. To validate the immune staining, external control slides were stained along with the lung carcinoma slides. Positive controls for PD-1, Nectin-2, and TIGIT were tonsil or other lymphatic tissue and tissue placental tissue for PD-L1 and PVR.

In order to standardize the staining, the H-Score was used [20], allowing the evaluation and comparison of the expression of each immune marker (PD-L1, PD-1, Nectin-2, PVR, and TIGIT). This score combines the intensity of staining, with values of one for weak, two for intermediate and three for strong staining intensity, and the area of positive staining which represents the tumor proportion score (TPS) of a particular section. These two variables are multiplied, resulting in a score-range from 0 to 300.

H−Score=stainingintensity×TPS

As the TPS applies for evaluating tumor cells we transferred this method on immune cells, reporting the proportion of positive stained immune cells on all available immune cells.

### Statistics

For statistical analyses of protein expression between the histological subtypes Mann-Whitney-U-Test was used, with a *P*-value of ≤ 0.05 considered statistically significant. Statistical analysis of correlation between different growth patterns and immunohistochemistry was performed by using R software and ‘ggcorrplot’ package (version 0.1.3) were exploited to figure out the data. Spearman correlation was used to compare protein expression. Venn diagram was calculated using R package ‘VennDiagram’ (version 1.6.20).

## Results

### Patients and tumor characteristics

Tumor samples from 22 lung adenocarcinoma patients resected at the University Medical Center Ulm were collected. All patients were diagnosed with NSCLC adenocarcinoma with a grading of I to III. The cohort included 9 male and 13 female patients, the age ranged from 42 to 79 years. For all patients staging was performed according to the IASLC UICC TNM (seventh edition) classification (Table 1).

**Table 1:** Baseline characteristics of patient cohort

	Age At Diagnosis	gender	grading	TNM-classification
Patient 1	42	f	I	T1a, Nx, L0,V0, R0, M0
Patient 2	48	f	II-III	n.a.
Patient 3	48	f	II	T2a, N2(10/12), Mx, L1, R1
Patient 4	54	f	I-II	T2b, N2 (N1 3/4; N2 3/15) M1a, R0
Patient 5	54	f	II-III	T2b, N1(7/7 N2), Mx, L1, V1, n0
Patient 6	58	f	II	T3, L0, V0, Pn0, Rx, M1b
Patient 7	59	m	II	T2a, N0, R0, M0
Patient 8	60	m	II	T4, N2 (N1 2/6, N2 17/34), L1, V0, Pn0, Mx, R0
Patient 9	59	f	III	T2a, N2 (N1 2/5, N2 2/11), L0, V1, Pn0, Mx, R0
Patient 10	60	m	II	T2, N0 (0/7), Mx, L0, V0, Pn0, R0
Patient 11	60	m	III	T2a, N2 (N1 2/9; N2 3/5), Mx, L1, V0, R0
Patient 12	62	m	I	T1a, N1 (N1 8/11; 0/2), Mx, L0, V0, R0
Patient 13	64	m	II	T4, V0, L0, N0 (N1 0/13; N2 0/15), Mx, R0
Patient 14	67	m	II	T1b, V0, L0, N0 (0/2), Mx, R0
Patient 15	66	m	I-II	T1b, V0, L0, N1 (hilar 1/4; N1 0/13; N2 0/3) Mx, R0
Patient 16	71	m	II	T1b, N2 (N1 3/7; N2 6/23), Mx, L1, V0, R0
Patient 17	74	f	II	T1b, N2, (N1 4/6; N2 2/3), Mx, R0, L0, V1, Pn0
Patient 18	75	f	II	T3, N0 (0/18), Mx, L0, V0, Pn0, R1
Patient 19	74	f	I	T2a, N2 (7/28), L0, V0, R0, M0
Patient 20	77	f	III	T1b, M0, R0, M0
Patient 21	78	f	I-II	T2, Nx, Mx, L0, V1, Pn0, Rx
Patient 22	79	f	II	n.a.

To examine the spatial distribution of the histological growth patterns and immune checkpoint proteins, up to 8 different tumor segments of each patient were assessed, with 20 patients providing 2 or more segments (min=1, max=8, mean=4), leading to a total of 84 segments (Table 2). Subsequently, immunohistochemical and histopathological examination were carried out separately for each of the 84 segments.

**Table 2:** Number of tumour segments per patient and register of every distinct growth pattern in each tumour segment

		Growth Patterns							
	Number of Segments	segment 1	segment 2	segment 3	segment 4	segment 5	segment 6	segment 7	segment 8
Patient 1	3	a, p, s	l	l, a, s					
Patient 2	4	a	a	a, s	a, s				
Patient 3	1	a							
Patient 4	5	l, a	a, p	a, p, s	l, a, p, s	l, a, p, s			
Patient 5	4	a	a, s	a, s	a, p, s, mp				
Patient 6	3	a, s	a, s	a, s					
Patient 7	4	a, s	a, s	a, s	a, s				
Patient 8	8	a, s, mp	p, s, mp	a, p, s	a, p, mp	a	a	a	a
Patient 9	2	l, s	s						
Patient 10	4	a, s	a, s	a, s	a, s				
Patient 11	2	l, a, s	l, a, s						
Patient 12	4	a, mp	a, p, s, mp	a, p, mp	a, p, s				
Patient 13	1	a, s							
Patient 14	5	a, s	s	s	s	s, mp			
Patient 15	3	p	p, mp	a, p, s, mp					
Patient 16	6	a, s	a, s	a, s	a, s	a, s	a, s		
Patient 17	5	a, s	a, s	a, s	a, s	s			
Patient 18	5	a	a	a	a	a			
Patient 19	4	l, a	l, a	l, a	l, a				
Patient 20	5	a, s	a, s	a, s, mp	a, s, mp	a, s, mp			
Patient 21	4	l, a, mp	l, a, mp	l, a	l, a				
Patient 22	2	a, p, s, mp	a, s, mp						
*total*	84	l=lepidic, a=acinar, p=papillary, s=solid, mp=micropapillary							

### Intra-heterogeneous spatial distribution of histopathological growth patterns

**Fig. 1:**
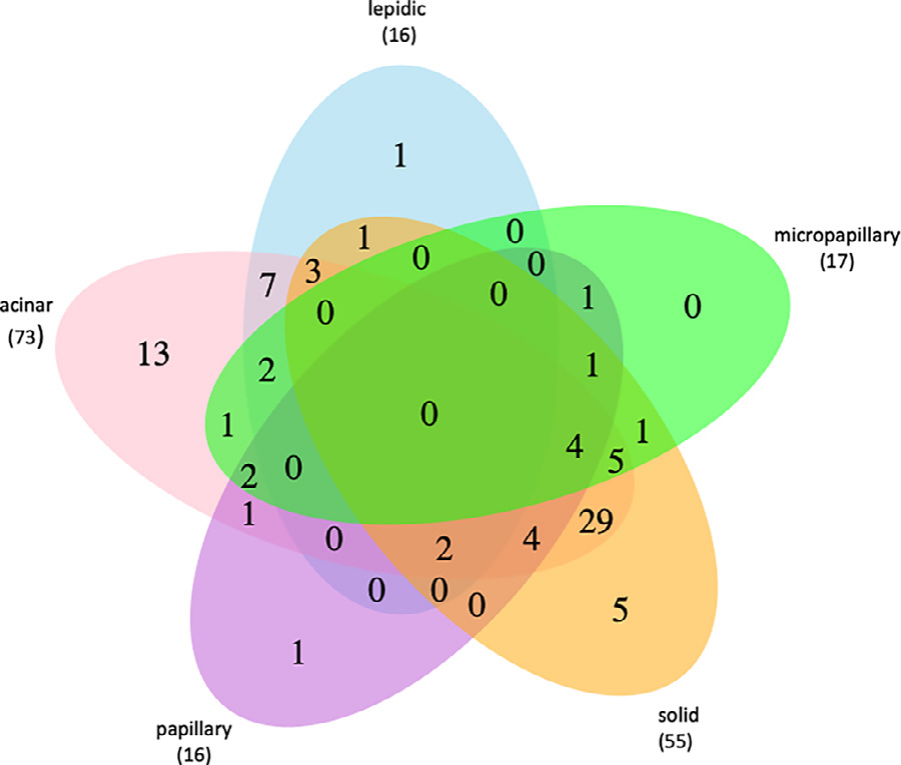
Venn diagram showing the occurrence of different growth patterns. Occurrence and combinations of lepidic, acinar, papillary, solid, and micropapillary growth patterns within all analysed tumour segments. The overall numbers are shown.

Each of the 84 tumor segments was examined histologically according to the World Health Organization classification guidelines for lung cancer 2015 [2], at which up to four different growth pattern areas per tumor segment were determined. This resulted in a total of 177 different growth pattern areas of which 73 (41%) were acinar, 55 (31%) solid, 16 (9%) papillary, 16 (9%) lepidic, and 17 (10%) micropapillary (Fig. 1).

Hence, 41 tumor segments harbored two different growth pattern areas, with acinar and solid being the most frequent combination (*n*=29). Further, 20 tumor segments harbored one exclusive growth pattern, with acinar being the most prominent (n=13). Moreover, 17 tumor segments harbored 3 different growth patterns and only six tumor segments harbored four different growth pattern areas, whereas in none of the segments all 5 growth patterns were observed (Fig. 1).

Two out of 22 patients (patient 3 and 18) had only one growth pattern, whereas nine had two (patient 2, 6, 7, 9, 10, 13, 16, 17, 19), four had three (11, 14, 20, 21) and seven had four (1, 4, 5, 8, 12, 15, 22) distinct growth patterns (Table 2).

Furthermore, in 20 patients more than two tumor segments were available. In 13 of those patients (65%) we observed single or multiple changes of growth patterns between the different segments (Table 2). However, only in seven patients (35%) the growth patterns did not vary between different segments.

### Heterogeneous spatial distribution of immune checkpoint protein expression

**Fig. 2:**
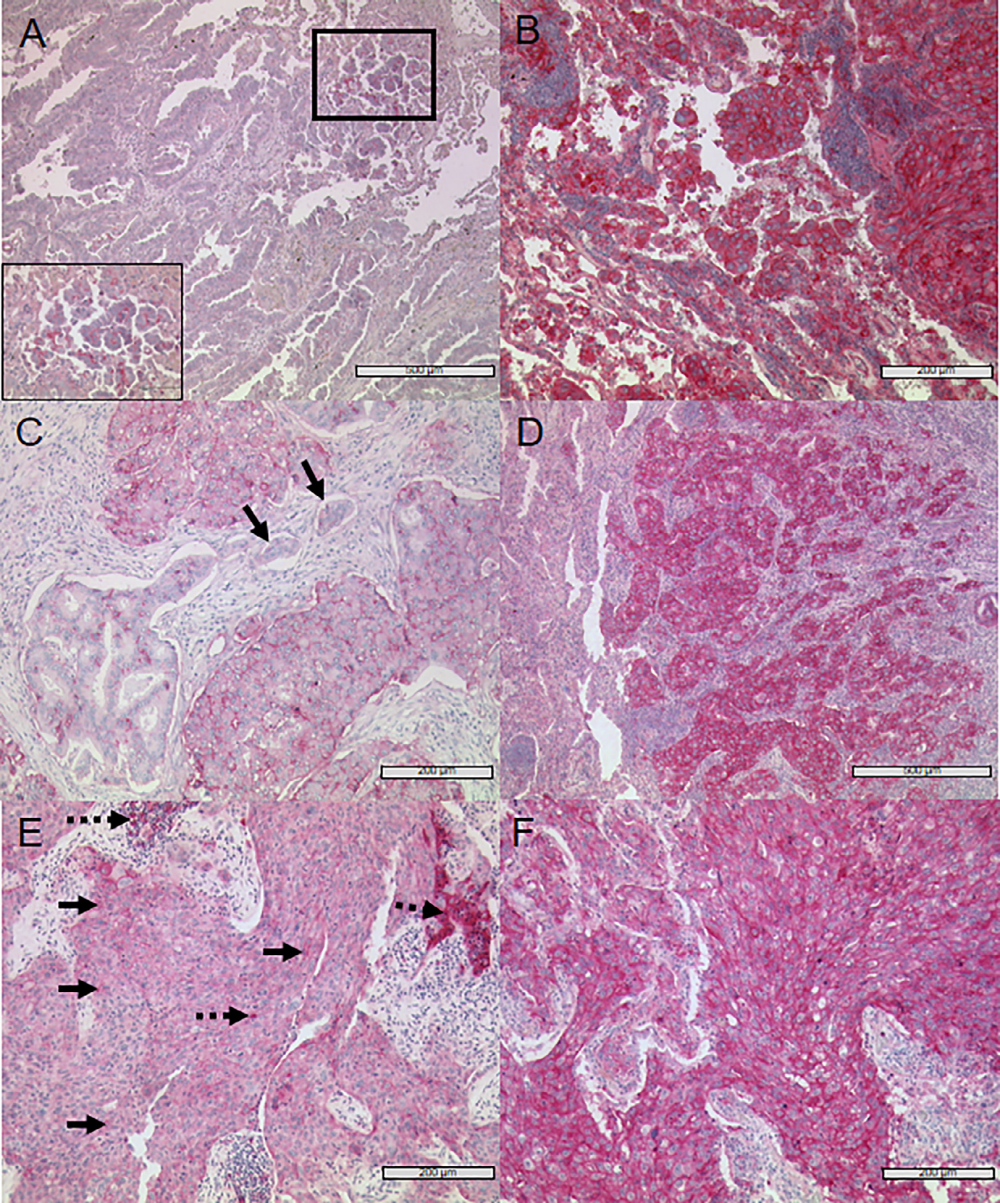
Representative images of immunohistochemical staining of lung adenocarcinoma with Nectin-2, PVR and PD-L1 (A) An area of micropapillary subtype weakly positive for Nectin-2 (see insert with higher magnification corresponding to marked area with thick lines) next to a Nectin-2 negative acinar growth pattern is shown. (B) Strong anti-Nectin-2 staining of acinar and solid growing tumour cells. (C) Solid subtype with weak anti-PVR staining and visible heterogeneity of protein expression on cell-level, aside negative/weaker acinar tumour cells and negative lymphangiosis cells (black arrows). (D) Strong positive PVR staining of solid growing tumour cells (*right*) whilst non-neoplastic tissue is negative (*left*). (E) Weak expression of PD-L1 on solid growing tumour cells (black arrows, continuous line) with groups of strongly PD-L1 positive TILs (black arrows, broken line). (F) Strong positive anti-PD-L1 staining of almost all tumour cells (solid growth pattern).

**Fig. 3:**
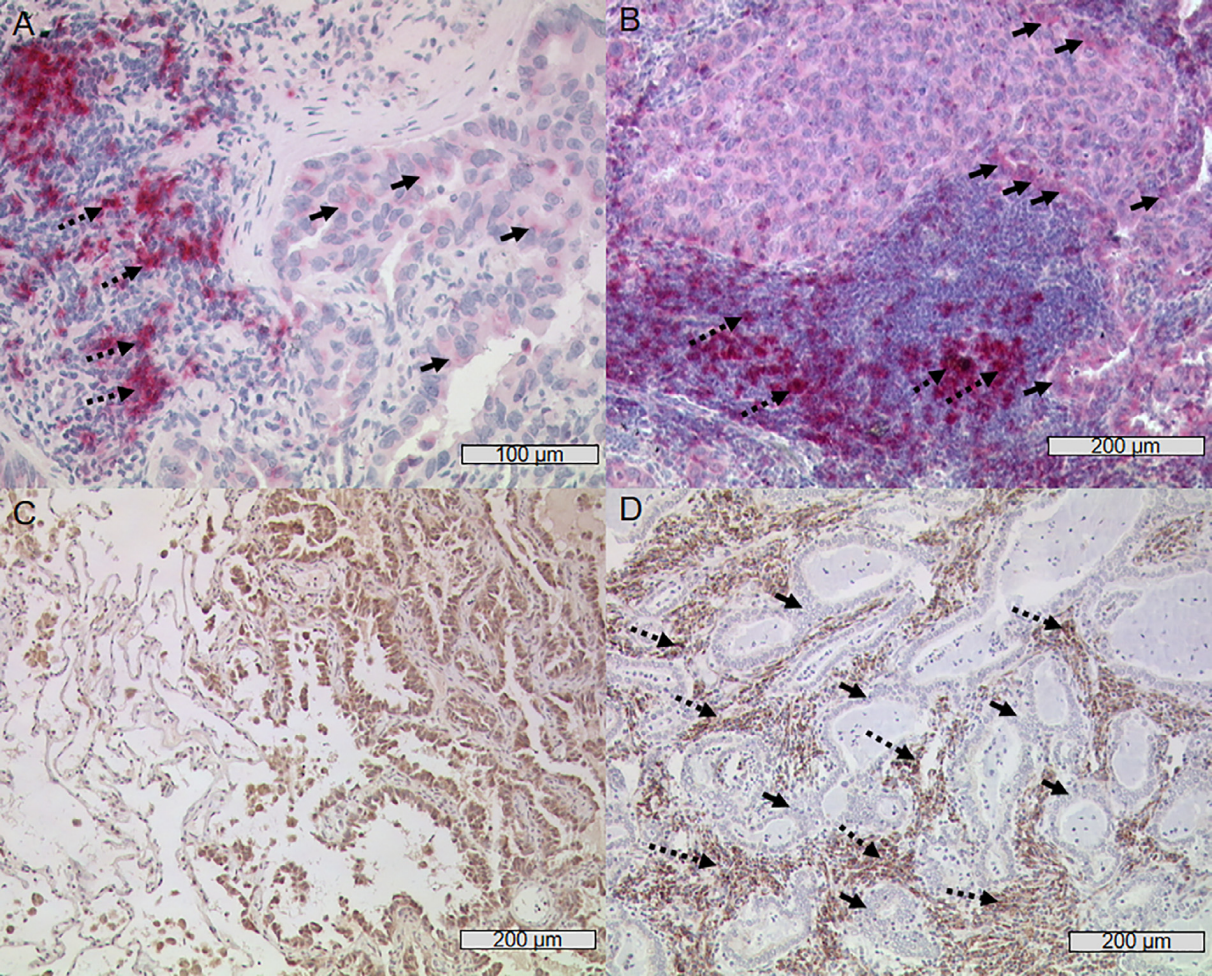
Representative images of immunohistochemical staining with PD-1 and TIGIT (A) Several strong PD-1 positive TILs (left, black arrows, broken line) next to single weakly stained tumour cells (right, black arrows, continuous line). (B) Weak PD-1 positive solid growing tumour cells (upper half of picture, black arrows, continuous line) and strong PD-1 positive TILs (lower half of picture, black arrows, broken line). (C) Non-neoplastic, TIGIT negative bronchial tissue cells (left) and strong TIGIT positive lung adenocarcinoma tumour cells with lepidic growth pattern (right). (D) Numerous strong TIGIT positive TILs (black arrows, broken line) infiltrating TIGIT negative highly differentiated acinar growing lung adenocarcinoma tumour (black arrows, continuous line).

After histological examination of the present growth patterns, immune checkpoint protein expression of PD-L1, PD-1, Nectin-2, PVR, and TIGIT was determined for all segments and growth pattern areas using IHC (Fig. 2, Fig. 3). We observed a high intra- and inter-tumoral heterogeneity of protein expression, regarding staining intensity and tumor proportion score (TPS) of the immune checkpoint ligands Nectin-2, PVR and PD-L1, as well as receptors PD-1 and TIGIT on tumor cells varied within one tumor segment, as well as between different tumor segments and growth pattern areas (Fig. 2 and 3). Therefore, this heterogeneity in marker expression was seen within single segments with areas of high expression in close proximity to areas with no or very low expression of the marker. Of note, the heterogenous expression was even observed on the level of single cells (Fig. 2).

Similar to the situation on tumor cells, we also observed a pronounced intra-tumoral heterogeneity of PD-1 and TIGIT on TILs (Fig. 3). Additionally, there was no expression of TIGIT in healthy bronchial tissue, but in the corresponding malignant tissue (Fig. 3C). Besides, an increase of TIGIT expression with further progression of dysplasia of the tumor cells was observed (Supplement 4). Furthermore, an adenocarcinoma pre-stage, i.e., the atypical adenomatous hyperplasia (AAH), was noticed to be TIGIT positive (Supplement 5).

### Distribution of immune checkpoint proteins in different growth patterns

**Fig. 4:**
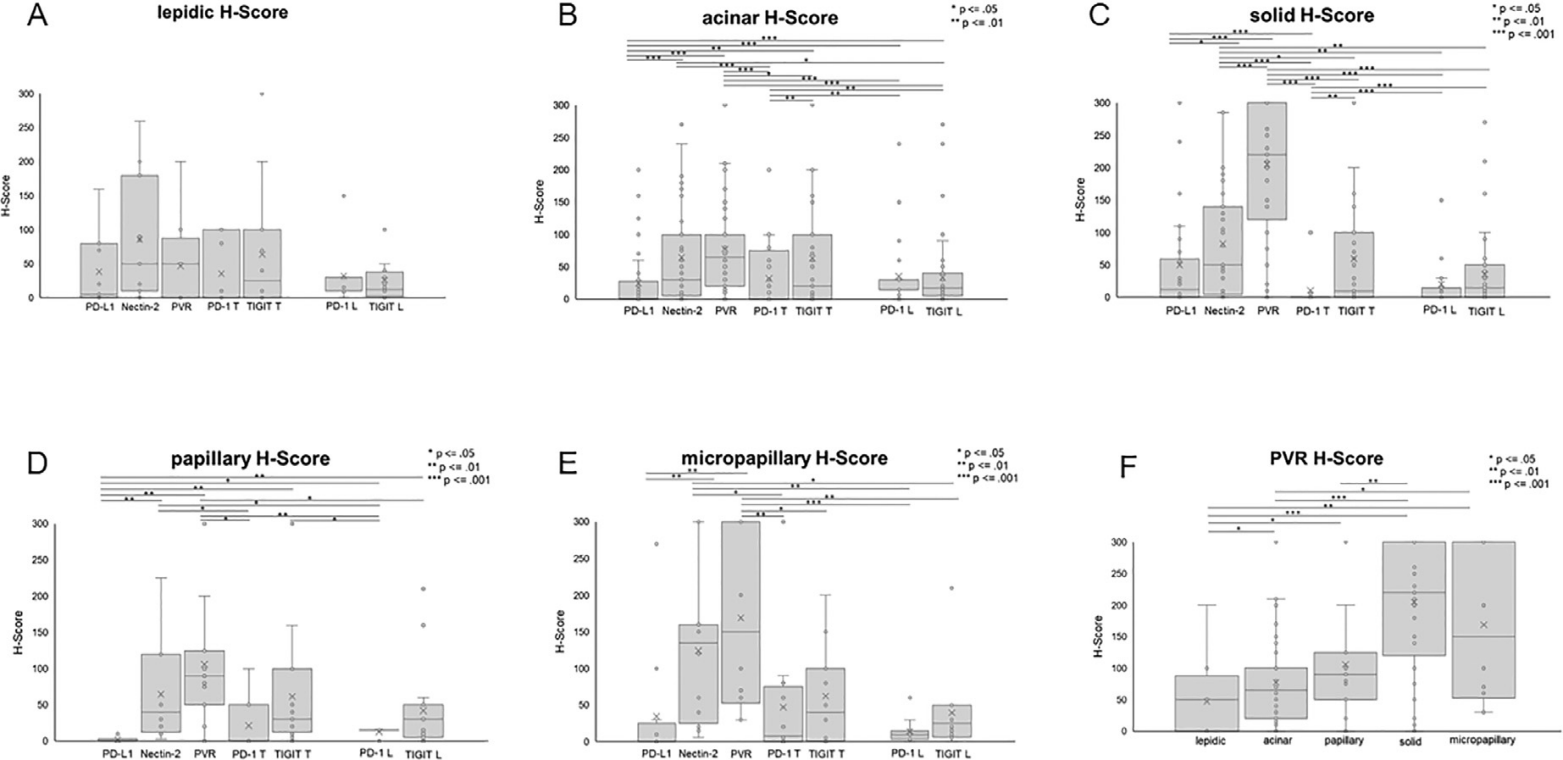
Immune checkpoint proteins display a high variety in different growth patterns. Expression of different immune checkpoint proteins in (A) lepidic, (B) acinar, (C) papillary, (D) solid, and (E) micropapillary growth patterns assessed using H-score. (F) Distribution of PVR in different growth patterns. Statistical significance: * = *P* ≤ 0.05, ** = *P* ≤ 0.01, *** = *P* ≤ 0.001. T = tumour, L = lymphocytes/TILs.

**Fig. 5:**
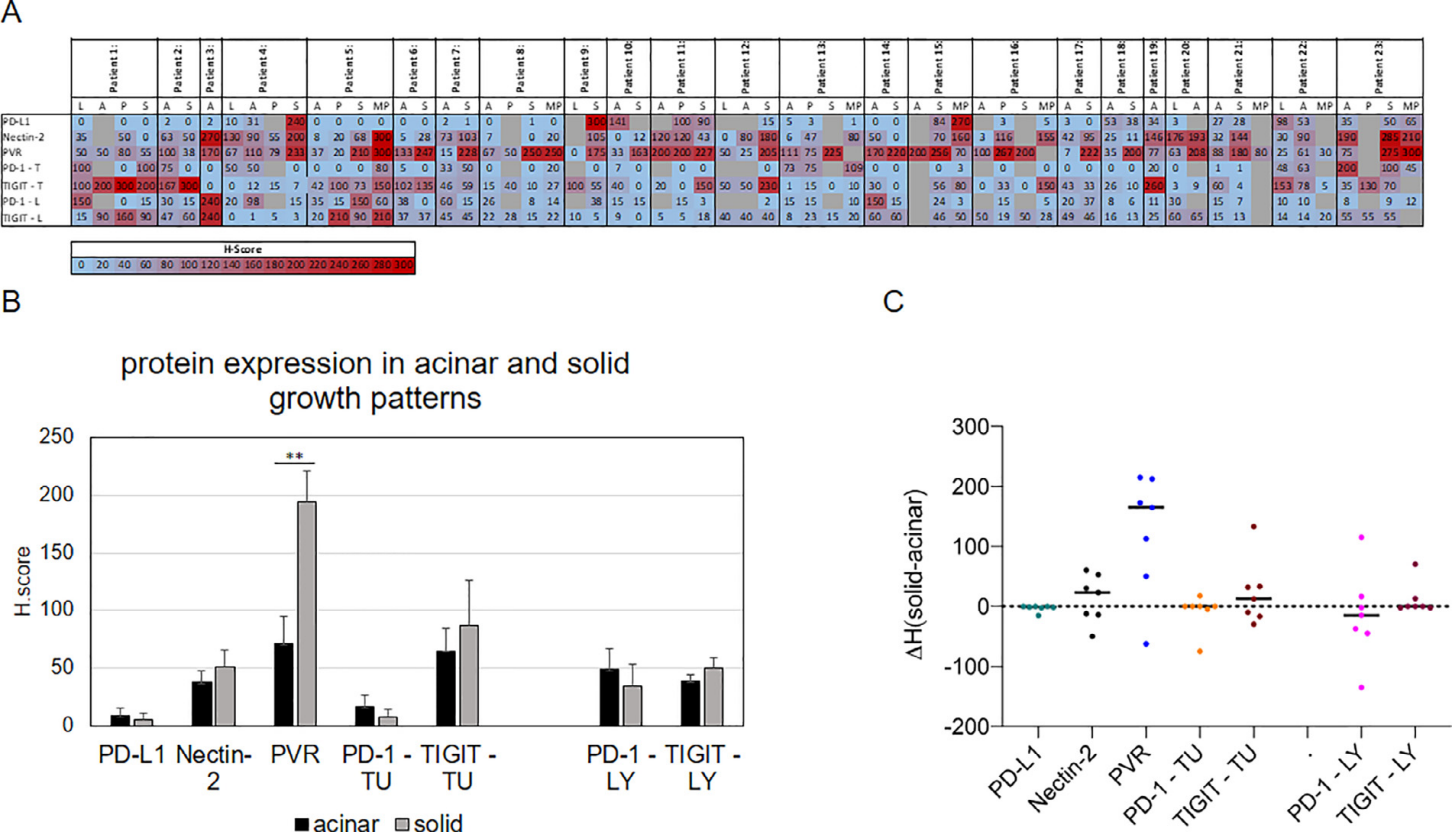
Immune checkpoint protein expression in different growth patterns of each patient. (A) Heatmap showing immune checkpoint protein expression in different growth pattern areas of each patient. H scores are given. (B) Comparison of immune checkpoint proteins in patients with acinar and solid growth patterns. Mean values and standard deviation are shown. Statistical significance: ** = *P* ≤ 0.01. (C) Difference of immune checkpoint protein expression between acinar and solid growth patterns in each patient. TU = tumour, LY = lymphocytes/TILs.

Comparing the expression of the analyzed immune checkpoint ligands and receptors, we observed a notable variation of the expression within one growth pattern, within one tissue segment as well as between the individual tumor patients (Fig. 4, Fig. 5).

Except the situation in the lepidic growth pattern, in which no statistical difference in the expression of the immune checkpoint proteins emerged, all other growth patterns displayed significant differences in the expression of the analyzed immune checkpoint proteins. In the acinar, solid, papillary, and micropapillary growth patterns, for instance, Nectin-2 and PVR were significantly higher expressed compared to PD-L1 (Fig. 4). Furthermore, the expression level of PVR exceeded the one of Nectin-2 in the solid growth pattern (Fig. 4C). PVR was also significantly higher expressed in papillary, solid and micropapillary growth patterns compared to the acinar and lepidic growth pattern (Fig. 4F).

We detected PD-1 and TIGIT expression on tumor cells in all morphological growth patterns, whilst in the acinar and solid growth patterns TIGIT expression on tumor cells was much higher than PD-1 expression on tumor cells (*P*= 0.00374 and *P*= 0.0012). In contrast, in none of the growth patterns a statistical difference was observed between PD-1 and TIGIT expression on TILS (Fig. 4).

Regarding the portion of immune checkpoint protein positive growth pattern areas, we observed a high variability. As an example, PD-L1 positive areas were more common in the lepidic (72,7%) and solid (61,8%) subtype than in acinar (53,6%), papillary (42,9%) or micropapillary (41,7%) (Supplement 2A).

### Co-occurrence of immune checkpoint protein expression within one patient

As co-occurrence of PD-1 and TIGIT expression was reported in NSCLC specimens, we analyzed the co-occurrence of the markers in the different growth pattern areas. As shown in Fig. 5, we observed a heterogenous picture regarding the co-occurrence of the analyzed immune checkpoint proteins.

All investigated immune checkpoint proteins (PD-L1, PD-1, Nectin-2, PVR, TIGIT) were expressed in 18 patients, whereas in 4 patients all immune checkpoint proteins except PD-L1 were expressed, suggesting a rather redundant than exclusive expression of the TIGIT:PVR/Nectin-2 system to the PD-1:PD-L1 axis. PD-1 and TIGIT on lymphocytes were expressed in all patients. Except for one patient (patient 3), TIGIT expression on tumor cells was observed in every patient and in 14 patients additionally PD-1 expression on tumor cells was examined (Fig. 5A).

In contrast, expression of PD-L1 was independent of PD-1 expression either on lymphoid cells or on tumor cells. Furthermore, in tumor segments of patients with a combination of solid and acinar morphological growth patterns, PVR was higher expressed in solid growth pattern compared to acinar growth pattern (*P*=0.00736, Fig. 5B). Further, regarding difference between H-Score obtained in acinar growth pattern and solid growth pattern of each patient, PVR is higher expressed in solid growth pattern compared to the acinar growth pattern of the same patient (Fig. 5C).

**Fig. 6:**
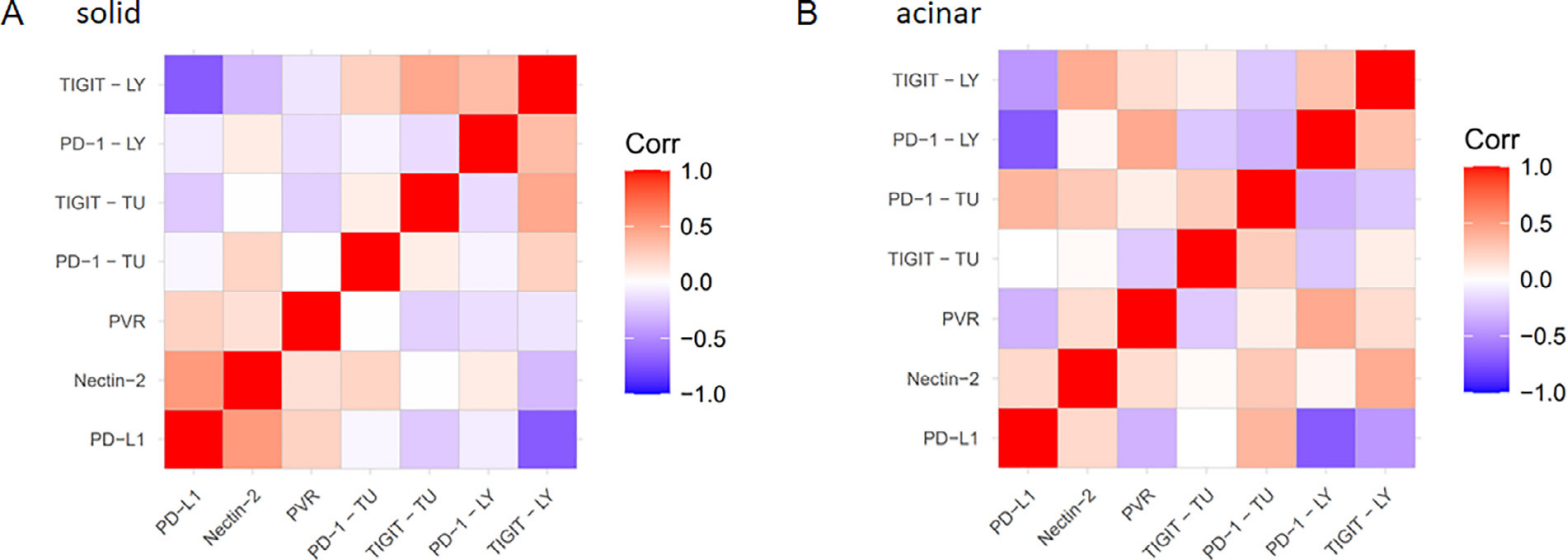
Correlation of protein expression within different growth patterns. (A) Correlation of immune checkpoint proteins in solid growth pattern. (B) Correlation of immune checkpoint proteins in acinar growth pattern. Blue colour marks a negative correlation, red colour marks a positive correlation. TU = tumour, LY = lymphocytes/TILs.

Moreover, we found no correlation between expression of different immune checkpoint proteins in general. However, when acinar and solid growth patterns were analysed separately, we obtained a positive correlation between Nectin-2 and PD-L1 expression (*Ρ*= 0.516; *P*= 0.02) in the solid growth pattern (Fig. 6A). In contrast, no significant correlation of the Nectin-2 and PD-L1 expression was observed in the acinar growth pattern (Fig. 6B). However, in the acinar growth pattern negative correlation between PVR and PD-L1 (*Ρ*= -0.327) was determined, though not being statistically significant (*P*= 0.114).

## Discussion

Immune checkpoint inhibitors represent a game changer in fighting cancer. Especially with the development of antibodies interrupting the PD-1:PD-L1 interaction, recent advancement of treatment for many tumor entities were obtained 21, 22, 23, 24, 25, 26, 27. However, what dampens the enthusiasm for this approach is the high rate of non-responders, which is up to 80% 28, 29, 30. Sardari Nia suggests that growth pattern classification represents a significant prognostic factor in NSCLC and therefore provides a possible explanation for survival differences [31,32]. In line with previous reports [4], we identified histological intra-tumoral heterogeneity in form of multiple growth patterns in almost every analyzed specimen. Only two out of 22 tumor specimens (patient 3 and 18) harbored one exclusive growth pattern, of which patient 3 contributed only one segment, suggesting this patient would also exhibit more growth patterns if more segments were available. Moreover, the ratio between the growth patterns in a given NSCLC tumor (specimen) is not fixed, as we observed a change in the growth pattern ratio between different segments of the same specimen. Furthermore, protein levels of PD-L1 and PD-1 varied immensely within and between individual segments, which is in accordance with previous reports, as they determined heterogeneous molecular profiles between different growth patterns 33, 34, 35, 36. Moreover, Cai et al. demonstrated intra-tumoral heterogeneity of EGFR and ALK status not only between different growth patterns, but also within growth patterns [36].

Another major cause for the failure in treatment response is discussed to be other existing immune checkpoint-pathways in the tumor microenvironment, probably being redundant to the PD-1:PD-L1 system. Hung et al. observed that overall survival in glioblastoma can be improved using a combination of anti-PD-1 treatment with other checkpoint inhibitors, for example anti-TIGIT [37]. Therefore, we investigated the protein levels of PD-L1, PD-1, Nectin-2, PVR, and TIGIT. We found a vast heterogeneity regarding these markers between different growth patterns and also within a given growth pattern. Furthermore, we demonstrated PVR being higher expressed in solid, papillary, and micropapillary growth pattern compared to lepidic and acinar growth pattern. Of note, up-regulation of PVR has been reported on a variety of different tumor cells, which leads to tumor invasion and progression and is known to be associated with worse outcome [38]. This is in line with lepidic growth pattern being prevalent in very early lesions and associated with favourable prognosis, whereas the solid growth pattern is preferentially present in higher tumor stages and is associated with recurrence and worse outcome [2,^4^,6]. Moreover, Sun et al. reported that CD155/PVR and TIGIT overexpression in lung adenocarcinoma is closely correlated with poor clinical outcome [18]. We examined both functionally redundant immune checkpoint proteins PVR and Nectin-2, as interactions between different ligands and receptors are possible, displaying the complexity of the TIGIT:PVR/Nectin-2 axis. Gorvel et al. suggested Nectin-2, which has a lower affinity to TIGIT compared to binding affinity of PVR to TIGIT, to be an interesting target in cases where PVR is weakly expressed [38]. However, our results show expression of PVR to be more common compared to Nectin-2.

When analysing immune checkpoint protein expression separately in solid growth pattern, a significant positive correlation between Nectin-2 and PD-L1 arose. This is in contrast to other findings, e.g., Lee et al. demonstrated a co-expression of Nectin-2 and PVR as well as an inverse correlation between PVR/Nectin-2 and PD-L1 using RNA sequencing and microarray data [39].

Further, we detected TIGIT and PD-1 expression on immune cells as well as on tumor cells. However, we observed no expression of TIGIT in non-neoplastic bronchial tissue, but in the corresponding malignant tissue nearby. This suggests that TIGIT expression is induced specifically in the tumor by yet unknown mechanisms. This notion is supported by the finding that AAH as a preinvasive early lesion of lung adenocarcinoma was also TIGIT positive. Similarly, an increased anti-TIGIT staining with progressive dysplasia of bronchial mucosa was observed. It is known that PD-1 is also expressed on tumor cells [40], however, TIGIT expression on tumor cells is only described on murine cell lines but not on human cells by now [41]. Therefore, to our best knowledge, our study is the first one to show the expression of TIGIT on human lung adenocarcinoma tumor cells.

With our findings we further illustrate the molecular and morphological inter- and intratumoral heterogeneity of lung adenocarcinoma. This systematic analysis confirms and expands the knowledge of heterogeneous spatial distribution of immune checkpoint proteins. Collectively, our results strongly imply that the situation in a small biopsy might not reflect the expression of the immune checkpoint proteins in the entire tumor. Thus, either a multiregion analysis of immune checkpoint proteins or an adjustment according to the given growth pattern is required in order to obtain a more realistic picture of the PD-1:PD-L1 and the TIGIT:PVR/Nectin-2 status as predictive markers for immune checkpoint inhibitor therapy.

## Appendix Supplementary materials

Image, application 1
