# Supplementary material for: Spatial distribution of immune checkpoint proteins in histological subtypes of lung adenocarcinoma
Source: Neoplasia. 2021 Jun 5;23(6):584–93. doi: 10.1016/j.neo.2021.05.005 (PMC8190489; doi:10.1016/j.neo.2021.05.005)
Supplement: Supplementary file 1 [file mmc1.docx]

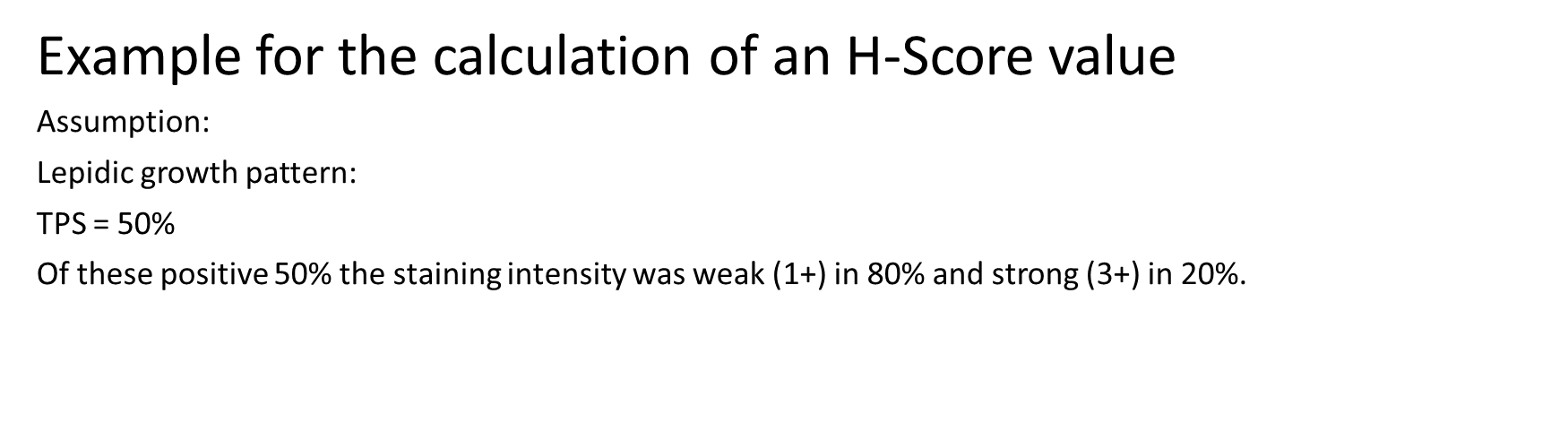
**Supplement 1. Example for the calculation of a H-score value**


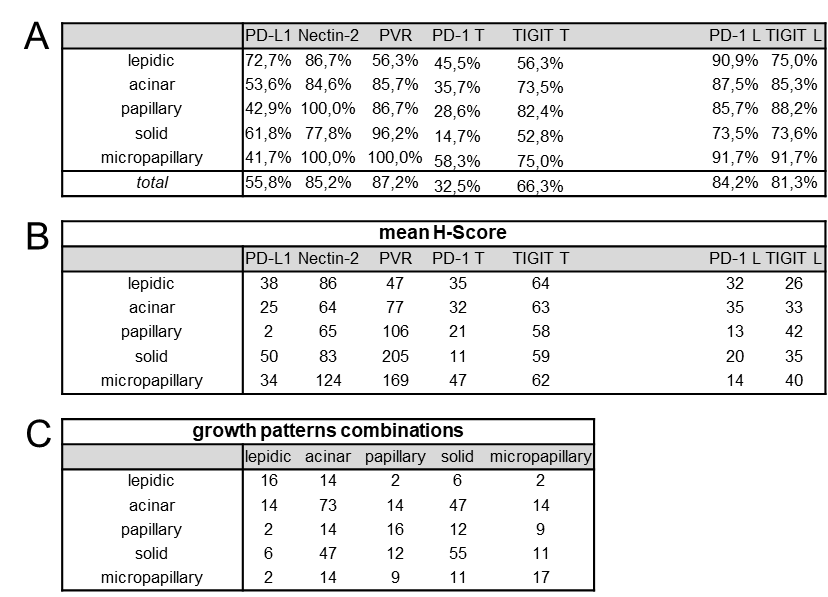


**Supplement 2. Summary of positive areas, mean H-score and growth pattern combinations.** (A) Percentage of PD-L1, Nectin-2, PVR, PD-1, and TIGIT positive areas in distinct growth patterns. (B) Mean H-Score of each protein for each growth pattern. (C) Incidence of growth pattern combinations.

**
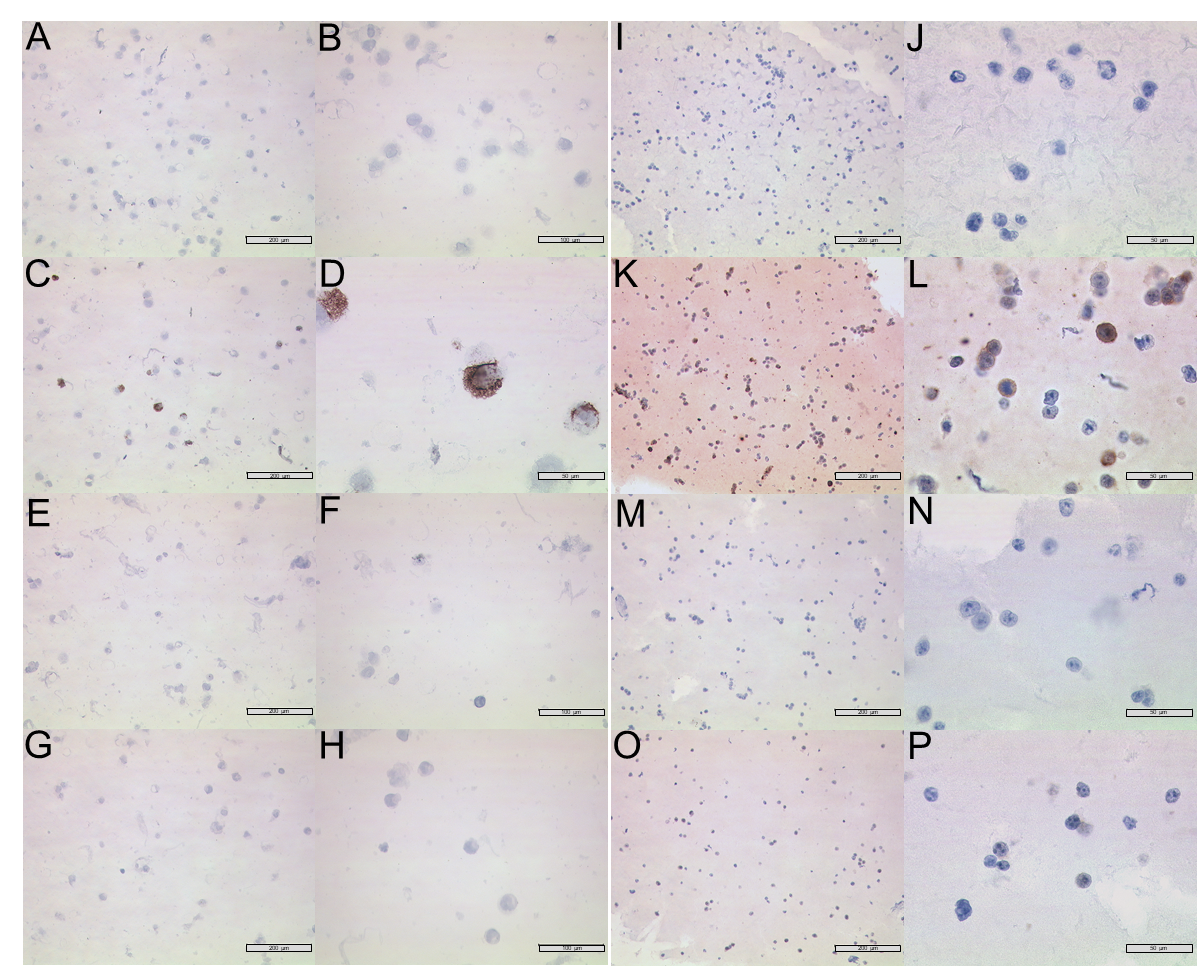
**

**Supplement 3. Validation of TIGIT staining: Ectopic expression of TIGIT in HEK293 cells.**

**IHC staining of cytospins (A-H), and sections of the cellblock (I-P) with the anti-TIGIT antibody TG1 (dianova).** (A), (B) Cytospin, (I), (J) section of cellblock of HEK293 cells transfected with pcDNA3.1 vector encoding human TIGIT cDNA, stained without primary anti-TIGIT antibody (negative control). (C), (D) Cytospin, (K), (L) section of cellblock of HEK293 cells transfected with pcDNA3.1 vector encoding human TIGIT cDNA, stained with anti-TIGIT (TG1) primary antibody. Image displays positive staining of the cells and therefore expression of the TIGIT protein. (E), (F) Cytospin, (M), (N) section of cellblock of HEK293 cells transfected with pcDNA3.1 vector (empty vector), stained without anti-TIGIT (TG1) primary antibody (negative control). (G), (H) Cytospin, (O), (P) section of cellblock of HEK293 cells transfected with pcDNA3.1 vector (empty vector), stained using the anti-TIGIT (TG1) primary antibody.

**
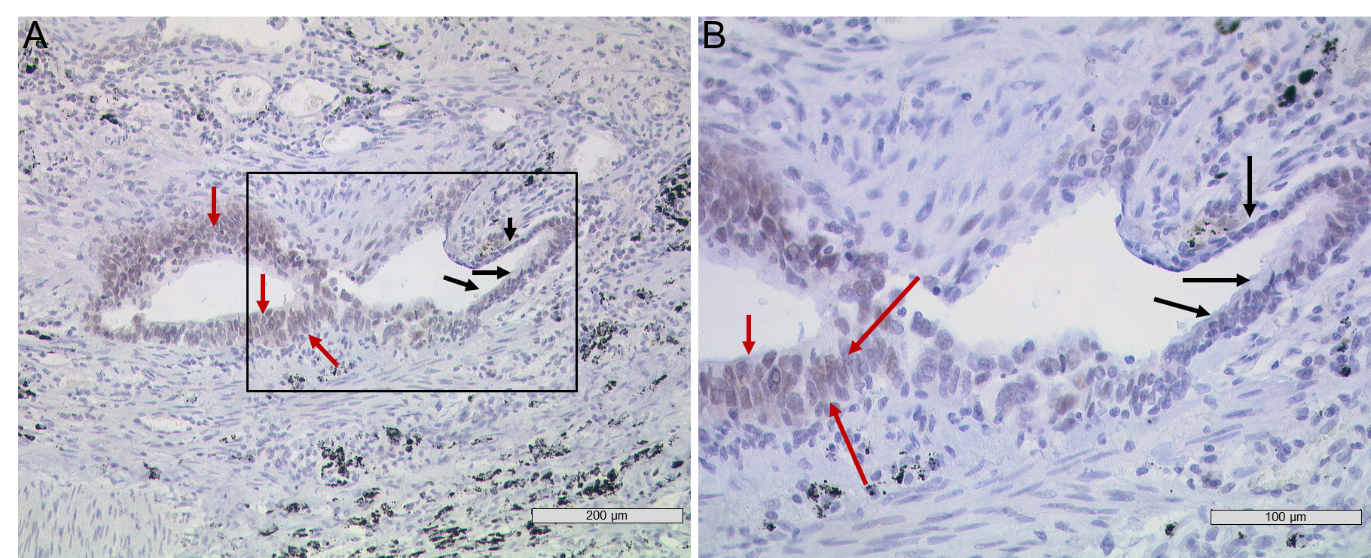
**

**Supplement 4. Increase of TIGIT expression with progressing dysplasia.** (A), (B) low grade dysplastic cells of a pulmonary bronchus are TIGIT negative (black arrows) whilst high grade dysplastic cells show weak TIGIT expression (red arrows). Side note: Anthrakosis.


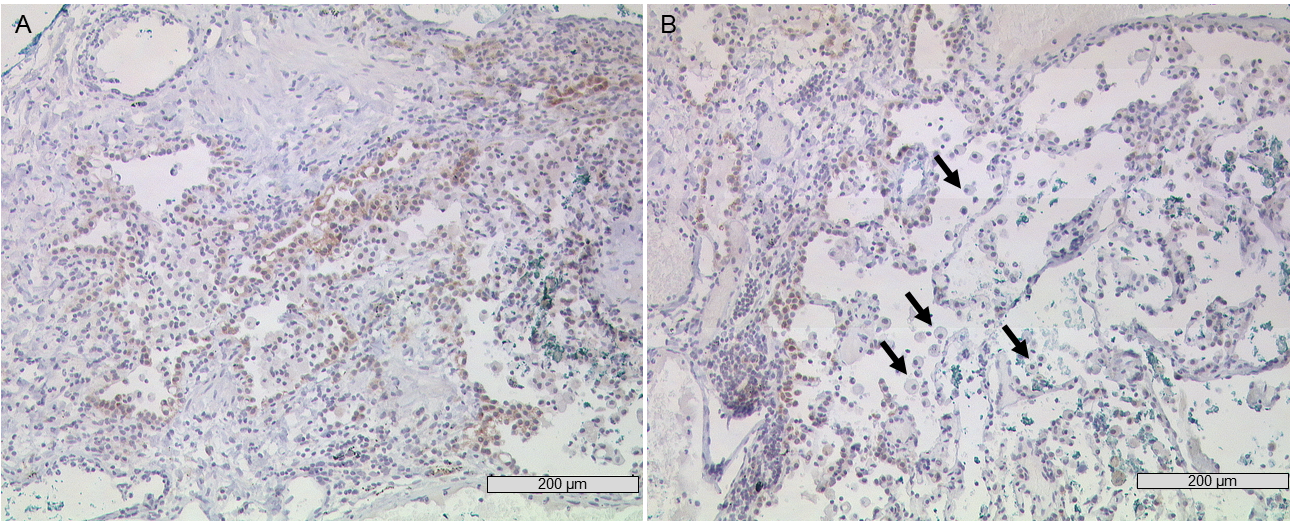


**Supplement 5.** (A) TIGIT positive preinvasive lesion of adenocarcinoma: atypical adenomatous hyperplasia (AAH), TIGIT positive AAH (left, center) next to negative non-neoplastic bronchial tissue (right) as well as negative alveolar macrophages (black arrows) (B)

**
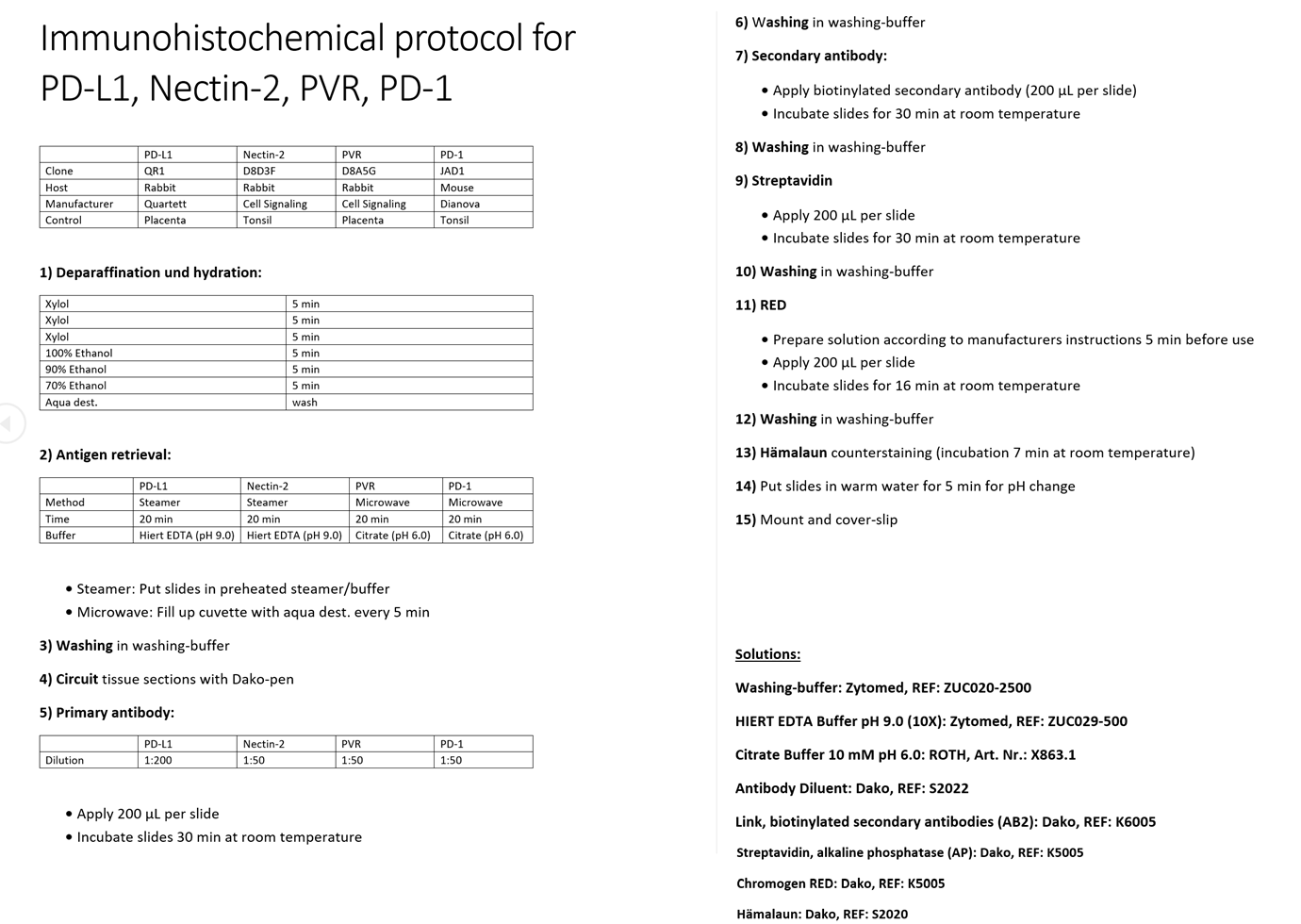
**

**Supplement 6. Immunohistochemical staining protocol for PD-L1, Nectin-2, PVR and PD-1.**

**
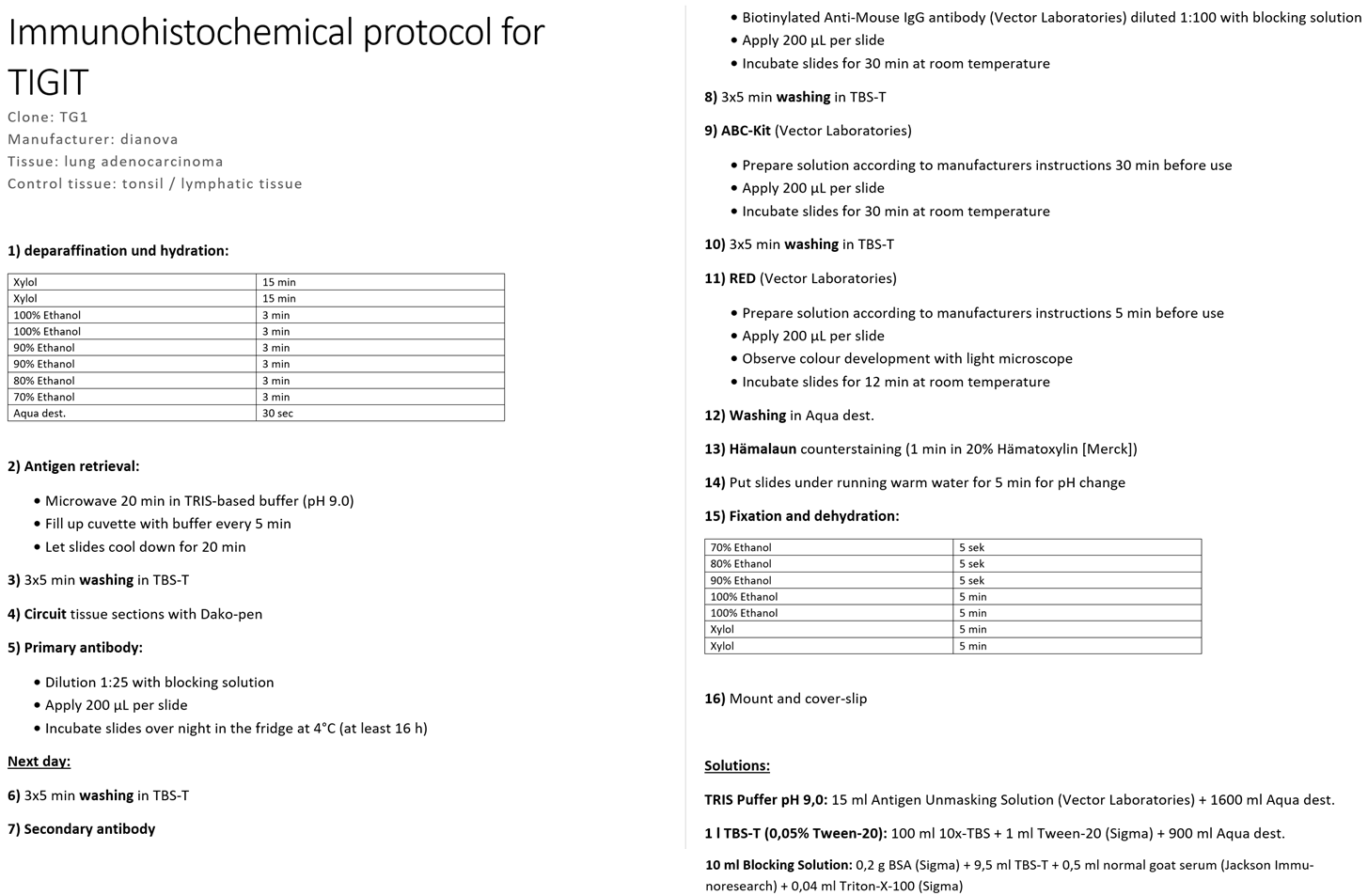
**

**Supplement 7. Immunohistochemical staining protocol for TIGIT.**
